# Understanding About Pesticide Handling Practice and Acute Pesticide Poisoning Among Farmers in Bangladesh

**DOI:** 10.1002/puh2.70219

**Published:** 2026-04-26

**Authors:** Md. Mejbah Uddin Mithu, Sadia Akter Shormela, Abdullah Jaman, Md. Shahinul Islam, Mahfuza Mubarak

**Affiliations:** ^1^ Department of Public Health Daffodil International University Savar, Dhaka Bangladesh; ^2^ Department of Public Health & Informatics Jahangirnagar University Savar, Dhaka Bangladesh; ^3^ Department of Public Health International University of Business Agriculture and Technology (IUBAT) Dhaka Bangladesh

**Keywords:** acute pesticide poisoning, Bangladesh, farmers, occupational exposure, pesticide use

## Abstract

The use of pesticides is very common in Bangladesh in order to improve the productivity of the agricultural sector, but due to its mishandling and lack of knowledge, there is a risk of exposure and consequent health effects among the farmers. The study was a cross‐sectional study that was carried out among 336 farmers in Naogaon district to determine the knowledge, practices, and health impacts associated with the use of pesticides. The data were collected with the help of face‐to‐face interviews with the use of structured questionnaire and analyzed within SPSS version 25. Chi‐square tests, correlation, and logistic regression analysis were done using descriptive statistics. The results have shown that the rate of unsafe pesticide behavior and lack of knowledge about the recommended practices is high. The age, education, farming experience, and the source of knowledge were also found to have a significant relationship with pesticide use behavior and health consequences. The number of farmers who complained of acute pesticide poisoning was very high. The article suggests that specific training and awareness campaigns as well as policy interventions are necessary to ensure the safe use of pesticides and minimize health hazards.

## Graphical abstract

Unsafe pesticide handling and limited awareness among Bangladeshi farmers lead to a high prevalence of acute pesticide poisoning and associated health problems. Strengthening training, awareness, and regulatory interventions is essential to promote safe pesticide practices and reduce occupational health risks.

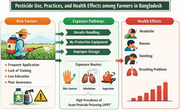

## 1 Introduction

As the world's population grows to 9 billion by 2050, food availability and accessibility become crucial challenges. Pesticides can help reduce pest output losses and meet this growing demand [1]. Modern farming uses pesticides, boosting output but causing environmental and public health problems due to widespread and careless use [2]. Pesticides are a chemical risk that is connected to food contamination, and pesticide contamination of food is a significant public health issue nowadays [3]. Pesticide exposure affects everyone; because of the additional danger of occupational exposure, agricultural workers are more prone to pesticide exposure [1]. The toxicity of a pesticide is determined by its function and other factors and can be assessed through methods like swallowing, breathing, or direct skin contact [4]. Food contamination by toxic chemicals poses a severe threat to public health, especially in Bangladesh where health literacy and awareness are extremely poor [5]. Pesticide‐contaminated vegetables have been shown to be hazardous to human health, especially when they are eaten raw [6]. Pesticide residues pose health risks, including headaches, nausea, vomiting, diarrhea, stomach pain, hypersensitivity, and endocrine disruption, particularly in children with small bodies and undeveloped immune systems [3]. Between 1997 and 2008, Bangladeshi farmers used 38% more toxic pesticides in agricultural areas, posing serious health risks due to long‐term residual effects [7]. Pesticides are believed to poison around 1.5 million farm workers annually, primarily in developing countries, with at least 20,000 dying from chemical exposure [7]. Farmers are overusing pesticides due to ignorance of residue risks, application timing, and regulations, leading to significant agricultural output issues [5]. Another author [8] The study highlights Ethiopian farmers’ underdevelopment, lack of reading skills, illiteracy, and ignorance about pesticide dangers, as well as their inability to use personal protective equipment (PPE) [9]. Farmers suffered from severe pesticide poisoning as a result of their lack of understanding about pesticide management [10]. The respiratory symptoms of farm laborers who used pesticides in Ethiopian fields included coughing, phlegm, and wheezing [11]. A study revealed 40% of Taiwanese fruit growers have pesticide contact allergies and 30% experience clinical dermatitis symptoms; 25.1% in Poland; and 12% in Japan [11]. Here, farmers received less advice and training about the precautions on the use of pesticides they should take to preserve the environment and themselves [10]. Bangladesh's agricultural sector has significantly impacted its GDP over the past decade, with a decrease from 17.6% in 2010 to 12.6% in 2020, with 16.5 million farmer families [12]. Farmers’ perceptions of pesticide exposure, lack of education, and inadequate knowledge about safe methods contribute to risk aggravation, necessitating higher education for better understanding and prevention [2]. Overall, 55% of agricultural laborers reported health issues, with respiratory, skin, gastrointestinal, conjunctivitis, arthritis, hypertension, diabetes, anemia, and hearing loss being the most common. Summer conditions include heat exhaustion and syncope [13].

The purpose of this study was to evaluate Naogan farmers’ levels of knowledge, attitudes, and behaviors regarding the responsible use of pesticides. The present study will be carried out to determine the knowledge and practice for the use of approved pesticides by the farmers and will investigate the residue contamination levels in vegetables collected from selected areas of Bangladesh. The study will be a scope to ensure food safety and build awareness about the impact of pesticides on human health with farmers and consumers.

## 2 Methodology

### 2.1 Study Area

**FIGURE 1:**
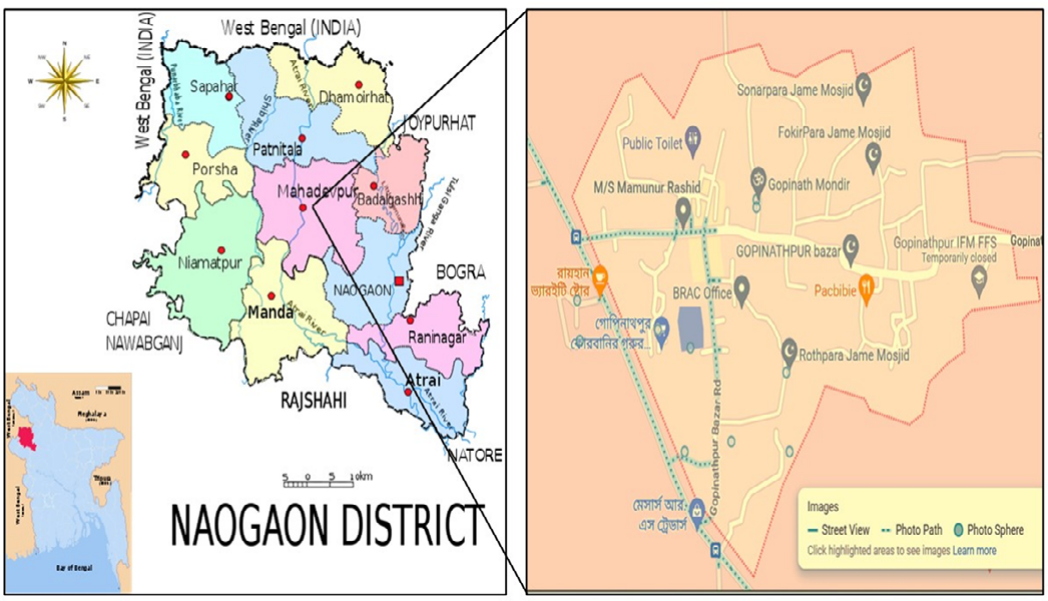
Map of study site.

This study was carried out in near the Naogoan district in Bangladesh, which lies within latitudes from 24°58′0.55″ N to 24°57′49.91″ N and longitudes from 89°5′4.65″ E to 89°5′11.76″ E, which is 500 km long (Figure 1).

### 2.2 Study Participants and Survey

Community‐based cross‐sectional study design was taken up to determine the knowledge and practices and the health outcome of pesticide use of the farmers. The design was chosen because it provides an opportunity to simultaneously measure exposure and outcome variables on the specified population. The study involved 336 farmers who are aged 18 years and above. The purposive sampling method was used in the selection of active farmers in Gupinathpur of Naogaon District. This strategy was selected because agricultural activities are concentrated in high density and respondents are easily available. Direct farmers who were directly engaged in the pesticide application were given precedence so as to make them relevant to the study objectives. The data were gathered using face‐to‐face interviews, and structured and pre‐tested questionnaire to enhance the accuracy of responses and reduce the chances of misunderstanding.

### 2.3 Measurements

Respondents’ understandings about pesticide handling process and acute pesticide poisoning (APP) were assessed using questionnaires. The questionnaire included three questions about dose, duration, and number of timings of pesticide use as well as clinical characteristics of the poisoning (i.e., primary symptoms and severity). All respondents could respond, “yes,” and “no.” The knowledge scores were calculated by assigning one point to each correct question, indicating more knowledge about pesticide handling practice and APP.

### 2.4 Statistical Analyses

Statistical examinations with IBM SPSS Statistics version 25 were used to make statistical analyses. The frequency and percent were used as descriptive statistics to summarize social‐demographic characteristics and pesticide use practices. To test the relationships between categorical variables (e.g., socio‐demographic variables and behavior of pesticide use) and health outcomes, the chi‐square test was used. This test was suitable in determining meaningful relationship between independent and dependent categorical variables. The strength and direction of relationships among the continuous or ordinal variables, in this case, the knowledge and pesticide dose accuracy, were evaluated using correlation analysis. The analysis of predictors of health issues and accuracy of pesticide dosage was based on binary logistic regression. The rationale behind the choice of this method is that the outcome variables were dichotomous (e.g., the presence or absence of health problems). The variables that were included in the regression model were chosen due to the previous literature and bivariate analysis that were statistically significant. The confidence interval of 95% (CI) was used, and *p* value lower than 0.05 was regarded as statistically significant.

## 3 Result and Discussion

The study surveyed 336 male farmers, with 75% being older than 40, are presented in Table 1. The majority were illiterate (72.9%), with only 11.9% and 15.2% having primary or secondary education. No farmer had attended higher education. The experience ranged from less than 10 years to over 30 years, with 44.9% having 21–30 years, 12.5% having more than 30 years, and 36.3% having 10–20 years of cultivating experience. According to [14] study, a study on the attitudes, knowledge, and practices of farmers in the Çukurova area regarding pesticide use found that all farmers were men, with 48.1% having been farming for over 20 years. Over half were over 40 years old, 59% completed high school or above, and only 1.4% were illiterate. In Lagos, Nigeria, 94.8% of respondents were men, with 50.7% aged 35–44. The majority had formal education, with 46.8% having secondary education and 6.5% having higher education. The majority had between 6 and 10 years of farming experience [15].

**TABLE 1:** Socio‐demographic status of farmers.

Variables	Frequency (*n* = 336)	Percent
**Age**		
<30	3	0.9
30–40	90	26.8
41–50	135	40.2
>50	108	32.1
**Educational status**		
Primary	40	11.9
Secondary	51	15.2
Illiterate	245	72.9
**Farming experience**		
<10	21	6.3
10–20	122	36.3
21–30	151	44.9
>30	42	12.5

Table 2, a study, shows 41–50 farmers believe increased pesticide use leads to higher crop yields, with 11.1% disagreeing. Educational background and farming experience influence perceptions, with 73.5% aware of pesticide rules [15]. A study in Lamatar, Nepal, found that 43.2% of agricultural workers were aware of pesticide use, with 73% aware of its environmental and human harm. Overall, 82.4% believed pesticides were necessary for production optimization [16]. A survey found 65.1% of farmers are unaware of pesticides’ negative effects but agree higher application improves crop productivity. 85.4% receive information from salespersons, and 92% believe pesticide use is linked to overcropping [16]. A study in Nepal found that 97% of 326 farmers, specifically 71.2% in Chitwan district, were knowledgeable about using pesticides daily [17]. A study in Punjab, Pakistan, revealed that 90% of well‐informed farmers believe more pesticide use is beneficial for increased crop production, with only 15% following the recommended schedule [18]. A study shows that pesticide usage is linked to perceived better crop output, with most farmers using pesticides 15 times to trim crops after blooming, despite only 2.9% knowing guidelines.

**TABLE 2:** Association between perceptions about pesticide use among variables.

	Do you think more use of pesticides is useful to production of more crops		Total	*X* ^2^ (*p* value)
	Yes	No		
**Age**				
<30 *n* (%)	0 (0.0%)	3 (100.0%)	3 (100.0%)	36.489^b^ (0.000***)
30–40 *n* (%)	75 (83.3%)	15 (16.7%)	90 (100.0%)	
41–50 *n* (%)	120 (88.9%)	15 (11.1%)	135 (100.0%)	
>50 *n* (%)	108 (100.0%)	0 (0.0%)	108 (100.0%)	
**Education**				
Primary *n* (%)	37 (92.5%)	3 (7.5%)	40 (100.0%)	165.195^a^ (0.000***)
Secondary *n* (%)	21 (41.2%)	30 (58.8%)	51 (100.0%)	
Illiterate *n* (%)	245 (100.0%)	0 (0.0%)	245 (100.0%)	
**Farming experience**				
<10 *n* (%)	12 (57.1%)	9 (42.9%)	21 (100.0%)	55.543^b^ (0.000***)
10–20 *n* (%)	99 (81.1%)	23 (18.9%)	122 (100.0%)	
21–30 *n* (%)	151 (100.0%)	0 (0.0%)	151 (100.0%)	
>30 *n* (%)	41 (97.6%)	1 (2.4%)	42 (100.0%)	
**Knowledge about the rules of pesticide use and its harmful aspects**				
Yes *n* (%)	86 (73.5%)	31 (26.5%)	117 (100.0%)	56.351^a^ (0.000***)
No *n* (%)	217 (99.1%)	2 (0.9%)	219 (100.0%)	
**Source of knowledge**				
Salesman *n* (%)	264 (92.0%)	23 (8.0%)	287 (100.0%)	45.758 (0.000***)
Any training program *n* (%)	1 (9.1%)	10 (90.9%)	11 (100.0%)	
Neighboring farmers *n* (%)	38 (100.0%)	0 (0.0%)	38 (100.0%)	
**Knowledge about use pesticide in a single day**				
Yes *n* (%)	295 (90.5%)	31 (9.5%)	326 (100.0%)	1.206^a^ (0.000***)
No *n* (%)	8 (80.0%)	2 (20.0%)	10 (100.0%)	
**Use pesticide in a single day**				
1 *n* (%)	22 (73.3%)	8 (26.7%)	30 (100.0%)	13.597^b^ (0.001***)
2 *n* (%)	237 (90.5%)	25 (9.5%)	262 (100.0%)	
3 *n* (%)	44 (100.0%)	0 (0.0%)	44 (100.0%)	
**Knowledge about government guidance of pesticide use**				
Yes *n* (%)	0 (0.0%)	10 (100.0%)	10 (100.0%)	94.635^a^ (0.000***)
No *n* (%)	303 (92.9%)	23 (7.1%)	326 (100.0%)	
**Number of times use pesticides after coming flowers to cut the crops**				
10 *n* (%)	34 (50.7%)	33 (49.3%)	67 (100.0%)	145.427^a^ (0.000***)
15 *n* (%)	266 (100.0%)	0 (0.0%)	266 (100.0%)	

*Note:* The confidence interval of 95% (CI) was used, and *p*‐value lower than 0.05 was regarded statistically significant.

The study in Table 3 shows a significant correlation between age, education, and health issues among Ugandan farmers, with higher education reducing prevalence, whereas illiteracy, elementary, and secondary education have the highest prevalence [19]. A survey shows 90% of 303 farmers believe pesticide use will increase crop production, but 97% have health issues, and 54.5% do not believe pesticides increase crop production [20]. A study on Kuwaiti farm laborers revealed that 80% of farmers believe pesticides are necessary for large crop yields, with 82% reporting APP symptoms, and the remaining 18% do not link health issues to pesticide exposure [2, 21]. A survey of Pakistani farmers revealed that despite facing health issues, their knowledge and usage of pesticides were significantly higher than their use of spraying machines [18]. Research in Badulla shows 13% of vegetable farmers spray insecticides, with 75% experiencing acute illness, whereas the remaining farmers have none [20]. A study reveals 88.9% of farmers experience health issues from pesticide use, with timing correlated. However, only 26.4% wear protective equipment, and 13.4% use PPE in China [22]. A study reveals 88.9% of farmers experience health issues from pesticide use, with timing correlated. However, only 26.4% wear protective equipment, and 13.4% use PPE in China [23]. In research by [9], a study in southeast Ethiopia found that 58.2% of farmers regularly use PPE, with masks, boots, gloves, and goggles being the most commonly used. In Pakistan, hand gloves, face masks, glasses, and boots or shoes were the most commonly used precautionary measures [18]. Mexican farmers use hand gloves, high shoes, safety goggles, and face masks but lack face masks, causing health issues. Only 4% meet PPE requirements during pesticide application [24]. A Lagos, Nigeria, survey revealed that despite health issues among 74.4% of farmers using water for hand washing, 99.5% used water alone, and 96% took a bath after pesticide use [16]. Uttarakhand farmers use soap and mud for hand cleaning after insecticide application, 72% take a bath, and 16% return to work [20].

**TABLE 3:** Association between experienced health problems due to pesticide use among variables.

	Facing health problems		*X* ^2^ (*p* value)
	Yes	No	
**Age**			
<30 *n* (%)	1 (33.3%)	2 (66.7%)	26.812^b^ (0.000***)
30–40 *n* (%)	77 (85.6%)	13 (14.4%)	
41–50 *n* (%)	126 (93.3%)	9 (6.7%)	
>50 *n* (%)	108 (100.0%)	0 (0.0%)	
**Education**			
Primary *n* (%)	37 (92.5%)	3 (7.5%)	79.276^b^ (0.000***)
Secondary *n* (%)	30 (58.8%)	21 (41.2%)	
Illiterate *n* (%)	245 (100.0%)	0 (0.0%)	
**Farming experience**			
<10 *n* (%)	13 (61.9%)	8 (38.1%)	45.033^b^ (0.000***)
10–20 *n* (%)	106 (86.9%)	16 (13.1%)	
21–30 *n* (%)	151 (100.0%)	0 (0.0%)	
>30 *n* (%)	42 (100.0%)	0 (0.0%)	
**Know the rules of pesticide use and its harmful aspects**			
Yes *n* (%)	95 (81.2%)	22 (18.8%)	36.799^a^ (0.000***)
No *n* (%)	217 (99.1%)	2 (0.9%)	
**Think about more use of pesticide is useful to production of more crops**			
Yes *n* (%)	294 (97.0%)	9 (3.0%)	80.982^a^ (0.000***)
No *n* (%)	18 (54.5%)	15 (45.5%)	
**Pesticide spraying equipment**			
Bucket *n* (%)	14 (82.4%)	3 (17.6%)	3.448 (0.244)
Spraying machine *n* (%)	296 (93.4%)	21 (6.6%)	
Bottle *n* (%)	2 (100.0%)	0 (0.0%)	
**Know how many times use pesticide in a single day**			
Yes *n* (%)	303 (92.9%)	23 (7.1%)	0.127^a^ (0.722)
No *n* (%)	9 (90.0%)	1 (10.0%)	
**Number of times to administrate pesticide in a single day**			
Single *n* (%)	23 (76.7%)	7 (23.3%)	12.478 (0.000***)
Twice *n* (%)	245 (93.5%)	17 (6.5%)	
Thrice *n* (%)	44 (100.0%)	0 (0.0%)	
**Timing of pesticide administration**			
**Early morning/Morning**			
Yes *n* (%)	128 (88.9%)	16 (11.1%)	5.983^a^ (0.014***)
No *n* (%)	184 (95.8%)	8 (4.2%)	
Afternoon			
Yes *n* (%)	42 (67.7%)	20 (32.3%)	72.305^a^ (0.000***)
No *n* (%)	270 (98.5%)	4 (1.5%)	
**Evening/At dusk**			
Yes *n* (%)	269 (100.0%)	0 (0.0%)	103.770^a^ (0.000***)
No *n* (%)	43 (64.2%)	24 (35.8%)	
**Use any protective things during using pesticides**			
Yes *n* (%)	73 (82.0%)	16 (18.0%)	21.428^a^ (0.000***)
No *n* (%)	239 (96.8%)	8 (3.2%)	
**Protective equipment's**			
**Hand gloves**			
Yes *n* (%)	6 (54.5%)	5 (45.5%)	6.426^a^ (0.01***)
No *n* (%)	67 (85.9%)	11 (14.1%)	
**High shoes**			
Yes *n* (%)	23 (79.3%)	6 (20.7%)	0.215^a^ (0.643)
No *n* (%)	50 (83.3%)	10 (16.7%)	
**Safety goggles**			
Yes *n* (%)	16 (64.0%)	9 (36.0%)	7.658^a^ (0.006***)
No *n* (%)	57 (89.1%)	7 (10.9%)	
**Face masks**			
Yes *n* (%)	35 (68.6%)	16 (31.4%)	14.535^a^ (0.000***)
No *n* (%)	38 (100.0%)	0 (0.0%)	
**Hand washing after use pesticide**			
Only water *n* (%)	207 (99.5%)	1 (0.5%)	52.539^b^ (0.000***)
With soap *n* (%)	67 (74.4%)	23 (25.6%)	
Ash + water *n* (%)	26 (100.0%)	0 (0.0%)	
Cly + water *n* (%)	12 (100.0%)	0 (0.0%)	

*Note:* The entire statistical analysis was done at a confidence interval (CI) of 95% (CI), and *p* value of less than 0.05 was regarded as statistically significant.

Table 4 shows pesticides pose health risks to humans, including acute and chronic poisoning, and can negatively impact agricultural workers in developing countries, causing respiratory issues, nausea, headaches, stomach pain, vomiting, and itching [15]. Farmers in Badulla face a variety of health issues, including headaches, skin issues, nausea, dizziness, vomiting, stomach discomfort, and eye problems [20]. Over half of farmers who handle pesticides experience skin issues, including vomiting, nausea, headaches, stomach pain, respiratory issues, muscular soreness, tears in eyes, burning, and itching. Most apply pesticides twice a day, with a significant correlation between the number and severity.

**TABLE 4:** Association between common health problems due to pesticide use and number of timings of administration in a single day.

	Number of times to administrate pesticide in a single day			*X* ^2^ (*p* value)
	Single	Twice	Thrice	
**Vomiting**				
Yes *n* (%)	15 (7.5%)	156 (78.4%)	28 (14.1%)	0.022^a^
No *n* (%)	8 (7.1%)	89 (78.8%)	16 (14.2%)	0.989
**Nausea**				
Yes *n* (%)	9 (45.0%)	11 (55.0%)	0 (0.0%)	26.283^b^
No *n* (%)	14 (4.8%)	234 (80.1%)	44 (15.1%)	0.000
**Headache**				
Yes *n* (%)	18 (26.5%)	45 (66.2%)	5 (7.4%)	47.522^a^
No *n* (%)	5 (2.0%)	200 (82.0%)	39 (16.0%)	0.000
**Stomach pain**				
Yes *n* (%)	13 (7.0%)	146 (78.5%)	27 (14.5%)	0.147^a^
No *n* (%)	10 (7.9%)	99 (78.6%)	17 (13.5%)	0.929
**Breathing problem**				
Yes *n* (%)	20 (37.0%)	31 (57.4%)	3 (5.6%)	60.520^b^
No *n* (%)	3 (1.2%)	214 (82.9%)	41 (15.9%)	0.000
**Muscle pain**				
Yes *n* (%)	3 (23.1%)	10 (76.9%)	0 (0.0%)	5.392^b^
No *n* (%)	20 (6.7%)	235 (78.6%)	44 (14.7%)	0.071
**Feeling sick**				
Yes *n* (%)	4 (40.0%)	6 (60.0%)	0 (0.0%)	10.041^b^
No *n* (%)	19 (6.3%)	239 (79.1%)	44 (14.6%)	0.009
**Tears in eye**				
Yes *n* (%)	5 (71.4%)	2 (28.6%)	0 (0.0%)	19.044^b^
No *n* (%)	18 (5.9%)	243 (79.7%)	44 (14.4%)	0.003
**Itching and burning of hands and feet**				
Yes *n* (%)	2 (9.5%)	18 (85.7%)	1 (4.8%)	1.642^b^
No *n* (%)	21 (7.2%)	227 (78.0%)	43 (14.8%)	0.485

*Note:* The entire statistical analysis was done at a confidence interval (CI) of 95% (CI), and *p* value of less than 0.05 was regarded as statistically significant.

**FIGURE 2:**
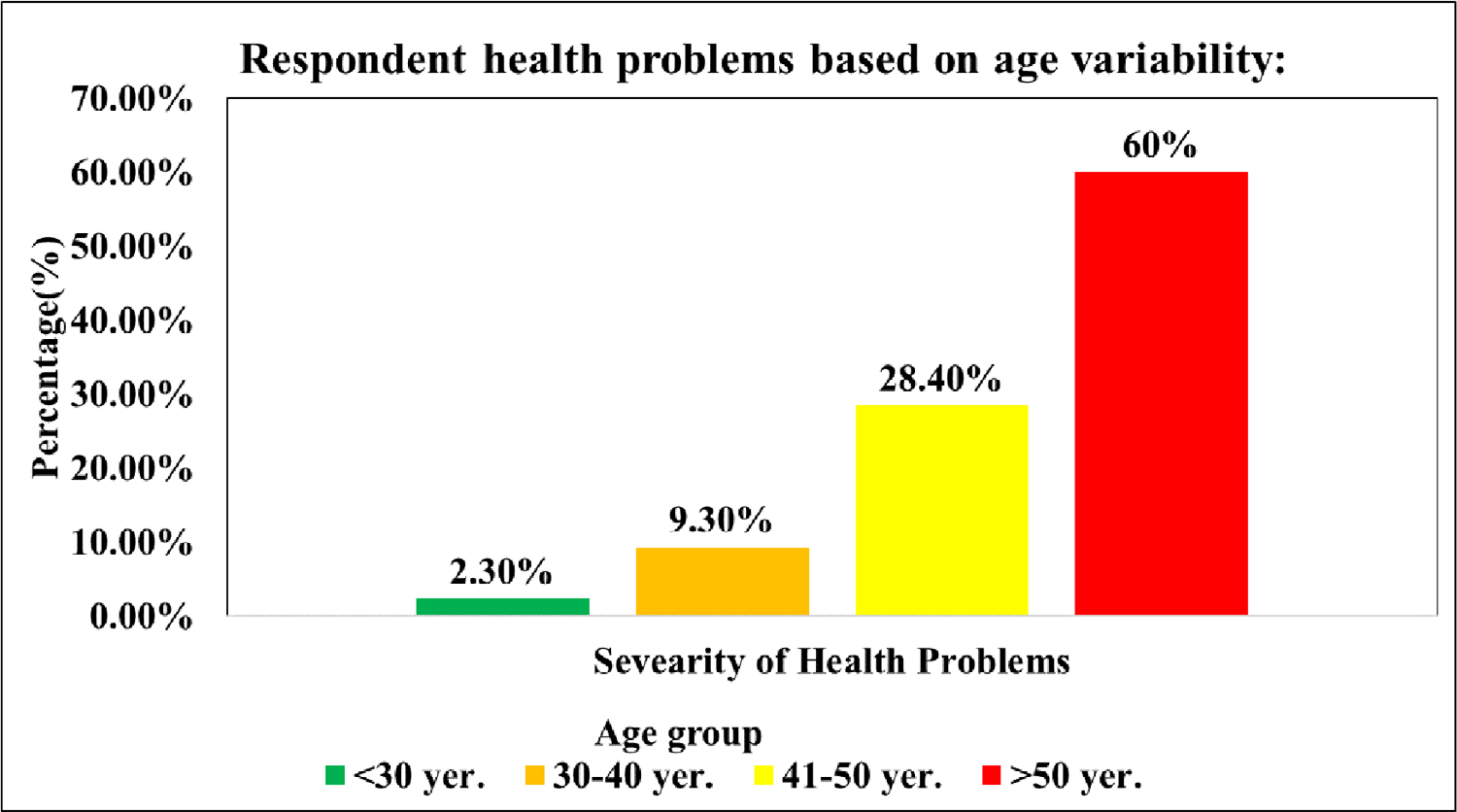
Health problems according to age.

Figure 2 depicts the association between age and health problems. It is evident that the prevalence of health issues is rising with age, with farmers between the ages of 20 and 25 experiencing health complications. Age groups are less than 30 years, 30–40 years, 41–50 years, and farmers above 50 years of age experiencing health difficulties were 2.3%, 9.3%, 28.40%, and 60%, respectively. Farmers over the age of 50 are the group with the highest prevalence of health complications (60%).

Table 5 shows the correlation between demographic variables and dose accuracy of pesticide use. Here, demographic variables, like age, educational status, farming experience, and having knowledge about pesticides, are considered independent variables, and dose accuracy is considered a dependent variable. According to the table, there is a positive correlation that has been detected. There is a positive moderate correlation between having knowledge and pesticide use dose accuracy. Here, *r* value is 0.441, and this correlation is also statistically significant as *p* value is 0.000, which is *p* < 0.05.

**TABLE 5:** Correlation between farmers’ pesticide dose use accuracy with demographic and existing knowledge source.

Sl. no.	Relationship	Range (±)	
1	Very weak	0.00–0.19	
2	Weak	0.20–0.39	
3	Moderate	0.40–0.59	
4	Strong	0.60–0.79	
5	Very strong	0.80–1.0	
Independent variable	Dependent variable	Coefficient of correlation (*r*)	*p* value
Age	Dose accuracy of pesticide	0.019	0.748
Education		0.008	0.911
Farming experience		−0.038	0.519
Knowledge source		0.441^**^	0.000

*Note:* The confidence interval of 95% (CI) was used, and *p*‐value lower than 0.05 was regarded statistically significant.

### 3.1 Binary Logistic Regression Analysis of the Predictive Variables of Health Conditions Among the Study Participants

The study used logistic regression to analyze the health conditions of farmers, and their pesticide use practices are shown in Table 6. The model included five independent variables: age, educational status, farming experience, and knowledge source. Two independent variables, years of farming experience, made a significant contribution to the model. Those with less than 10 years of farming experience were less likely to report health problems. Age groups 41–50 and above 50 years were also less likely to report health problems.

**TABLE 6:** Logistic regression predicting likelihood of reporting health problems.

Variables	*p* value	OR	95% CI	
			Lower	Upper
**Age**	0.069			
<30 years	0.553	0.398	0.019	8.361
30–40 years	0.130	0.054	0.001	2.357
41–50 years	0.039	0.010	0.000	0.794
>50 years	0.033	0.014	0.000	0.712
**Education**	0.451			
Primary	0.792	0.659	0.030	14.654
Secondary	0.308	0.167	0.005	5.207
Illiterate	0.342	0.245	0.014	4.443
**Experience**	0.046	0.071	0.005	0.954
**Knowledge source**	0.263	3.292	0.409	26.470

*Note:* The confidence interval of 95% (CI) was used, and *p*‐value lower than 0.05 was regarded statistically significant.

A binary logistic regression model is used in Table 7 to examine the impact of age, educational status, farming experience, and source of knowledge on respondents’ accurate pesticide dosage knowledge. Results showed that personal experience was less likely to lead to a strong understanding of pesticide dosage.

**TABLE 7:** Logistic regression predicting likelihood of reporting a pesticide dose use accuracy knowledge.

Variables	*p* value	OR	95% CI	
			Lower	Upper
**Age**	0.312			
<30 years	0.662	0.746	0.201	2.774
30–40 years	0.777	0.895	0.417	1.923
41–50 years	0.549	0.765	0.319	1.836
>50 years	0.031	0.254	0.073	0.885
**Education**	0.093			
Primary	0.068	7.000	0.866	56.564
SSC	0.376	2.625	0.310	22.233
HSC	0.154	4.449	0.572	34.629
**Experience**	0.518	1.253	0.632	2.485
**Knowledge source**	0.002	0.147	0.043	0.505

*Note:* The confidence interval of 95% (CI) was used, and *p*‐value lower than 0.05 was regarded statistically significant.

## 4 Recommendation

Department of agricultural extension must be used to carry out targeted training on the safe handling of pesticides. Pesticide dependency can be decreased by strengthening farmers’ field schools and encouraging the use of integrated pest management (IPM) practices. The Bangladesh food safety authority should conduct regular monitoring, policy enforcement, and awareness campaigns as a result of which the health risks will be reduced and food safety will be ensured.

## 5 Conclusion

The article has shown that unsafe pesticide handling habits and insufficient knowledge level among farmers are very common in Bangladesh and contribute to the APP and associated health issues. The socio‐demographic variables (age, education, and farming experience; pesticides lack formal training) are important determinants of pesticide use behavior. The results highlight the necessity of organized training programs, tight regulation, and community‐based awareness efforts to facilitate safe pesticide use. Health risk can be minimized and occupational safety enhanced significantly through the institutional reinforcement of support and incorporation of farmer education into agricultural extension services. Safe use of pesticides is the way of ensuring health protection of both the farmers and the people in general.

## Author Contributions

**Md. Mejbah Uddin Mithu**: conceptualization, methodology, data collection, formal analysis, writing – original draft. **Sadia Akter Shormela**: data curation, investigation, writing – review and editing. **Abdullah Jaman**: statistical analysis, validation, software. **Md. Shahinul Islam**: supervision, methodology, critical revision of the manuscript. **Mahfuza Mubarak**: project administration, supervision, final approval of the manuscript.

## Funding

The authors have nothing to report.

## Disclosure

This research was fully self‐funded. All laboratory equipment and analysis costs were borne by the authors.

## Consent

Informed consent was obtained from all individual participants included in the study. Prior to data collection, participants were informed about the purpose of the research, confidentiality of their responses, and their right to withdraw at any time without any consequences. Only those who voluntarily agreed were included in the study.

## Conflicts of Interest

The authors declare no conflicts of interest.

## Data Availability

The datasets generated and analyzed during the current study are available from the corresponding author upon reasonable request.

## References

[1] M. P. Ali , M. M. M. Kabir , S. S. Haque , et al., “Farmer's Behavior in Pesticide Use: Insights Study From Smallholder and Intensive Agricultural Farms in Bangladesh,” Science of the Total Environment 747 (2020): 141160, 10.1016/J.SCITOTENV.2020.141160. 32781314

[2] M. F. A. Jallow , D. G. Awadh , M. S. Albaho , V. Y. Devi , and B. M. Thomas , “Pesticide Knowledge and Safety Practices Among Farm Workers in Kuwait: Results of a Survey,” International Journal of Environmental Research and Public Health 14, no. 4 (2017): 340, 10.3390/ijerph14040340. 28338612 PMC5409541

[3] S. Begum , S. Sultana , M. S. Ahmed , and M. A. K. Azad , “Pesticide Residue Analysis From Winter Vegetables Collected From Six Markets of Rajshahi Bangladesh,” Journal of Environmental Science and Natural Resources 12, no. 2 (2019): 43–50.

[4] FAO/WHO . Pesticide Residues in Food Joint FAO/WHO Meeting on Pesticide Residues (FAO/WHO, n.d.).

[5] M. Hasan and A. Rahman , “Pesticide Residues in Selected Vegetable Collected From Wet Markets of Bangladesh,” Advances in Social Sciences Research Journal 6, no. 5 (2019): 15–23, 10.14738/assrj.65.6494.

[6] S. Akomea‐Frempong , I. W. Ofosu , E. D. G. J. Owusu‐Ansah , and G. Darko , “Health Risks Due to Consumption of Pesticides in Ready‐to‐Eat Vegetables (Salads) in Kumasi,” Ghana International Journal of Food Contamination 4, no. 1 (2017): 1–11, 10.1186/s40550-017-0058-6.

[7] M. S. Islam , “Farm Level Pesticides Use in Patuakhali and Comilla Region of Bangladesh and Associated Health Risk,” Journal of Health and Environmental Research 2, no. 4 (2016): 20, 10.11648/j.jher.20160204.11.

[8] M. Shammi , A. Sultana , N. Hasan , et al., “Pesticide Exposures Towards Health and Environmental Hazard in Bangladesh: A Case Study on Farmers' Perception,” Journal of the Saudi Society of Agricultural Sciences 19, no. 2 (2020): 161–173, 10.1016/j.jssas.2018.08.005.

[9] H. A. Gesesew , K. Woldemichael , D. Massa , and L. Mwanri , “Farmers Knowledge, Attitudes, Practices and Health Problems Associated With Pesticide Use in Rural Irrigation Villages, Southwest Ethiopia,” PLoS ONE 11 (2016): e0162527, 10.1371/journal.pone.0162527. 27622668 PMC5021266

[10] S. Bhattacharjee , M. Chowdhury , A. Fakhruddin , and M. Alam , “Impacts of Pesticide Exposure on Paddy Farmers' Health,” Jahangirnagar University Environmental Bulletin 2 (2013): 18–25, 10.3329/jueb.v2i0.16326.

[11] C. N. Kesavachandran , M. Fareed , M. K. Pathak , V. Bihari , N. Mathur , and A. K. Srivastava , “Adverse Health Effects of Pesticides in Agrarian Populations of Developing Countries,” in Reviews of Environmental Contamination and Toxicology (Springer Science & Business Media, 2009), 10.1007/978-1-4419-0028-9_2. 19680610

[12] Data collection for agriculture census begins, (Dhaka Tribune, (2019), 2009), https://www.dhakatribune.com/business/178997/data‐collection‐for‐agriculture‐census‐begins.

[13] H. Ahm , H. Mt , I. Ms , and A. Fs , “Common Health Problems Among Agricultural Workers in A Selected Rural Area of Mymensingh, Bangladesh,” CBMJ 11, no. 02 (2022): 125–130.

[14] D. Öztaş , B. Kurt , A. Koç , M. Akbaba , and H. Ilter , “Knowledge Level, Attitude, and Behaviors of Farmers in Çukurova Region Regarding the Use of Pesticides,” BioMed Research International 2018 (2018): 6146509, 10.1155/2018/6146509. 30112406 PMC6077575

[15] A. A. Alex , N. K. Longinus , A. M. Olatunde , and N. V. Chinedu , “Pesticides Related Knowledge, Attitude and Safety Practices Among Small‐Scale Vegetable Farmers in Lagoon Wetlands,” Journal of Agriculture and Environment for International Development 112, no. 1 (2018): 81–99, 10.12895/jaeid.20181.697.

[16] S. Jyoti , A. Yadav , S. Vaidya , and J. Sk , “Knowledge, Attitude, and Practice of Pesticide Use Among Agricultural Workers of Lamatar, Lalitpur, Nepal,” International Journal of Occupational Safety and Health 13, no. 4 (2023): 441–449, 10.3126/ijosh.

[17] S. Kafle , A. Vaidya , B. Pradhan , E. Jørs , and S. Onta , “Factors Associated With Practice of Chemical Pesticide Use and Acute Poisoning Experienced by Farmers in Chitwan District,” Nepal International Journal of Environmental Research and Public Health 18, no. 8 (2021): 4194, 10.3390/ijerph18084194. 33920994 PMC8071468

[18] S. Afsheen , “A Cross Sectional Survey of Knowledge, Attitude and Practices Related to the Use of Insecticides Among Farmers in Industrial Triangle of Punjab, Pakistan,” PLoS ONE 16 (2021): e0255454, https://go.gale.com/ps/i.do?id=GALE%7CA672601056&sid=googleSch. 34411142 10.1371/journal.pone.0255454 PMC8376108

[19] A. H. Oesterlund , J. F. Thomsen , D. K. Sekimpi , J. Maziina , A. Racheal , and E. Jørs , “Pesticide Knowledge, Practice and Attitude and How It Affects the Health of Small‐Scale Farmers in Uganda: A Cross‐Sectional Study,” African Health Sciences 14, no. 2 (2014): 420–433, 10.4314/ahs.v14i2.19. 25320593 PMC4196420

[20] M. A. Sachintha Prasad Jayasinghe and K. Thirumarpan , “Perception and Practices Towards Pesticides and Associated Health Impacts Among Vegetable Farmers in Badulla,” Journal of Agricultural Sciences—Sri Lanka 15, no. 2 (2020): 173–187, 10.4038/jas.v15i2.8798.

[21] A. Kumar , S. Pugazhendi , C. Kumar , J. Davidson , and J. Rawat , “Knowledge and Practices Regarding Pesticide Application and Handling Among Farmers in Selected Community Areas of Uttarakhand,” International Journal of Research in Medical Sciences 9, no. 4 (2021): 1187, 10.18203/2320-6012.ijrms20211373.

[22] C. Zhang , R. Hu , J. Huang , et al., “Health Effect of Agricultural Pesticide Use in China: Implications for the Development of GM Crops,” Scientific Reports 6 (2016): 34918, 10.1038/srep34918. 27721390 PMC5056523

[23] M. P. De‐Assis , R. C. Barcella , J. C. Padilha , H. H. Pohl , and S. B. F. Krug , “Health Problems in Agricultural Workers Occupationally Exposed to Pesticides,” in Revista Brasileira de Medicina do Trabalho (Associacao Nacional de Medicina do Trabalho, 2021), 10.47626/1679-4435-2020-532. PMC7879472 33597986

[24] J. Blanco‐Muñoz and M. Lacasaña , “Practices in Pesticide Handling and the Use of Personal Protective Equipment in Mexican Agricultural Workers,” Journal of Agromedicine 16, no. 2 (2011): 117–126. 21462024 10.1080/1059924X.2011.555282

